# Rare Atypical Ela3 BCR-ABL transcript in acute Lymphoblastic Leukemia: a case report

**DOI:** 10.4314/ahs.v25i2.41

**Published:** 2025-06

**Authors:** Lingling Xu, Tingting Han, Shuning Wei, Ningning Wang

**Affiliations:** 1 Department of Hematology, Yantai Yuhuangding Hospital Affiliated to Qingdao University, Yantai, China; 2 Peking University People's Hospital, Peking University Institute of Hematology, Beijing Key Laboratory of Hematopoietic Stem Cell Transplantation, Beijing, China; 3 Leukemia Center, institute of Hematology and Blood Diseases Hospital, Chinese Academy of Medical Sciences, Tianjin, China

**Keywords:** Acute lymphoblastic leukemia, atypical BCR-ABL transcript, ela3, tyrosine-kinase inhibitor, case report

## Abstract

The Philadelphia chromosome is usually express on about 30% acute B lymphoblastic leukemia. Most of Ph-positive acute lymphoblastic leukemia patients have ela2 BCR-ABL transcripts, other atypical fusion genes such as ela3 have been rare reported. We reported a case of Ph-positive B-acute lymphoblastic leukemia with a scare ela3 fusion transcript. She presented with a complex karyotype and showed good early response to imatinib and dasatinib but relapsed six months after diagnosis, and E255v, T315I mutations were successively detected in the ABL kinase region, then he switched to ponatinib and underwent allogeneic hematopoietic stem cell transplantation. But the Minimal Residual Disease increased after 16 months, the patient was treated with CD19 chimeric antigen receptor T cell immunotherapy, and changed to olverembatinib targeted therapy. This subgroup of acute lymphoblastic leukemia might have poorer prognosis than patients with common transcripts. we recommend the third-generation tyrosine-kinase inhibitor as a first choice for their initial therapy and allogeneic hematopoietic stem cell transplantation or immunotherapy and new clinical trials should be considered as early as possible.

## Introduction

Acute lymphoblastic leukemia (ALL) is a common malignant tumor of the blood system. It is mainly characterized by the abnormal proliferation of naive lymphocytes, which infiltrate the bone marrow or other tissues and organs, causing abnormal hematopoietic function and immune dysfunction. It usually includes two subtypes: acute B lymphoblastic leukemia and acute T lymphoblastic leukemia. The Philadelphia chromosome (Ph), referred to as t(9;22)(q34;q11.2), is the reciprocal translocation between chromosome 9 with the ABL gene and chromosome 22 with the BCR gene, which resulting in the BCR-ABL fusion gene^1^. It has been demonstrated that more than 95% chronic myeloid leukemia (CML) and 30% adult acute B lymphoblastic leukemia (B-ALL) can express this gene. The ph-positive ALL shows a poor outcomes and application of the tyrosine kinase inhibitor (TKI) in treatment can improve the prognosis^2^. The breakpoints in the ABL gene usually occur at exon a2, but in the BCR gene, there are three main types has been observed: the M-bcr which breakpoints cluster region usually located in exons e12-e16(also known as b1-b5), e13a2(b2a2) and e14a2(-b3a2), encoding a 210-KDa protein and mainly seen in CML; the m-bcr, ela2, encoding a 190-KDa protein and usually associating with B-ALL; the u-bcr, e19a2, encoding a 230-KDa protein and mainly causes mature neutrophils hyperplasia^3^,^4^. About 70% of Ph-positive ALL patients have ela2 BCR-ABL transcripts, and about 25% of cases have b2a2 and b2a2 transcripts. however, other atypical fusion genes such as ela3, e6a2 have been rare reported. Because of the variant forms are infrequently, the clinical characteristics and prognosis remain uncertain. Here we describe a case of adult Ph-positive common B-ALL with a scare ela3 fusion transcript.

## Case Presentation

**Figure 1:**
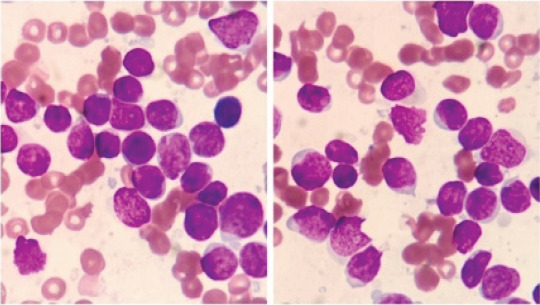
BM cell morphology with hematoxylin-eosin staining at high magnification (100×) of the patient at the time of diagnosis, which showed a large number of immature cells

**Figure 2:**
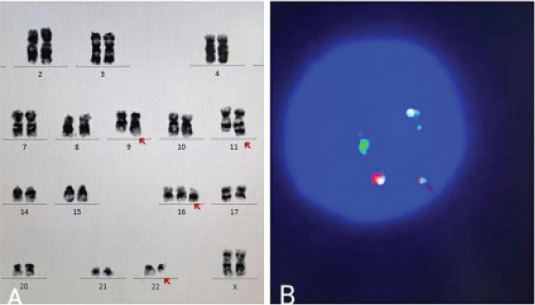
The patient's chromosome results showed a complex karyotype, and Philadelphia chromosome was presented(A). The red-green superimposed signals were detected by FISH, which confirmed the fusion of BCR (green) and ABL (red) genes(B)

**Figure 3:**
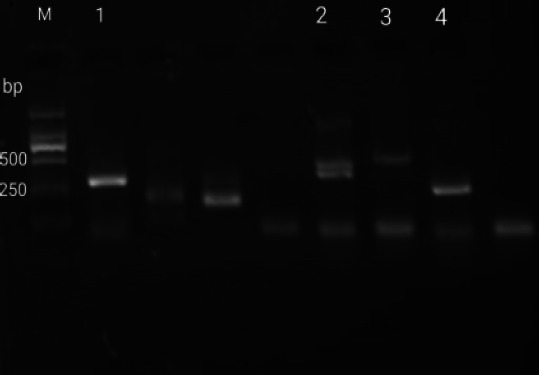
Electrophoresis results of BCR-ABL fusion gene detected by RT-PCR; M: Marker; 1: Target gene; 2: positive control p210; 3: positive control p190; 4: positive control p230

**Figure 4:**
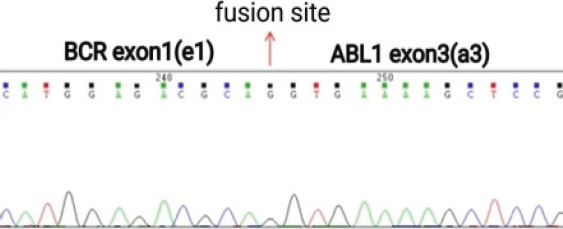
Sequencing analysis revealed the e1a3 BCR-ABL1 fusion transcript in patient

A 56-year-old female was admitted to hospital in September 3, 2020 for leukocytopenia and anemia. She reported no history of genetic disease or malignancy. Computed tomography (CT) scan revealed no space-occupying lesions. The blood test showed the white blood cell (WBC) count was 1.2×109 L-1 with 68% lymphoblasts, the hemoglobin (HGB) level was 91g/L and the platelet (PLT) count was 157×109 L-1, the bone marrow aspiration was promptly performed and showed an hypercellular infiltration of 78.5% blast cells (Figure 1) which also existed in 10% peripheral blood. A fresh specimen from the bone marrow aspirate was collected for molecular and cytogenetic testing. Flow cytometry immunophenotypic analysis demonstrated these abnormal cells strongly expressed CD10, CD19, cCD79a, CD34, TDT, HLA-DR, but no MPO, sIgM, cIgM or other T cells and granulocyte markers, and all these findings were consistent with a B-lymphoblastic leukemia. The karyotype analysis showed 46,XX,t(9:22)(q34:q11.2),?dup(11)(q13q25)[10]/46,idem,+16[6]/46,XX[4]. However, the initial BCR-ABL gene test was negative which was not consistent with the presence of Philadelphia chromosome. We considered the possibility of atypical BCR-ABL fusion gene, and the fluorescence in situ hybridization (FISH) confirmed it (Figure 2). Reverse transcription-polymerase chain reaction (RT-PCR) was used to detect fusion gene. According to2.5% agarose GEL electrophoresis, an atypical amplified band of 300 base pairs (bp) was identified, smaller than the P190 (ela2) fragment (Figure 3). In order to find the specific fusion site, we carried out a next generation sequencing, and it finally revealed the presence of the e1a3 variant (Figure 4).

Above all, the patient was diagnosed with Ph-positive common B-ALL. Then she started to induction chemotherapy with imatinib (400 mg/d), cyclophosphamide, daunorubicin, vincristine and prednisolone referring to the Treatment Guideline for China Adult Acute Lymphocyte Leukemia, and achieved complete response by morphology and immunophenotype, the BCR-ABL gene quantification decreased to 1%, and the chromosomal karyotype returned to normal, but a new gene mutation E255V was detected in the ABL kinase region which was moderately sensitive to dasatinib and resistant to imatinib. Therefore, the patient switched to dasatinib (100 mg/d) combined with CAM (cyclophosphamide 750 mg/m2d1, 8; cytarabine 100 mg/m^2^ d1-2, 8-9; 6-mercaptopurine 100 mg/d d1-7) scheme chemotherapy. The patient received three-drug (methotrexate, cytarabine, dexamethasone) intrathecal injections during each treatment to central nervous system prophylaxis.

After two courses of CAM regimen chemotherapy, the patient was treated with dasatinib monotherapy instead of combination chemotherapy for personal reasons. Unfortunately, the patient's disease relapsed in March 2021, and another gene mutation T315I appeared in the ABL kinase region, and it was resistant to dasatinib, so the patient altered to ponatinib (45 mg/d) therapy. After one month, the patient achieved complete remission again, and the BCR-ABL quantification decreased to 0. On May 25, 2021, the patient underwent allogeneic hematopoietic stem cell transplantation (allo-HSCT) from an HLA-identical sibling donor at Peking University People's Hospital. However, after 16 months, the patient underwent CAR-T therapy due to MRD elevated, and changed to olverembatinib targeted therapy. There were no obvious adverse reactions during the whole treatment. At the time of writing this report, the patient was still alive, but had not yet achieved complete molecular remission.

## Discussion

The production of fusion genes causing by chromosome ectopic is one of the most important pathogenesis of leukemia. It can encode oncoproteins with constitutive tyrosine kinase activity, and alter the proliferation rate, survival signal, immunology-related effects and microenvironment of hematopoietic stem cells^5^. The BCR-ABL fusion gene is a significant molecular diagnostic marker for CML and ALL. Among the three main fusion types, b3a2 and b2a2 types are present in 83.0% to 90.4% CML and about 25% ph-positive ALL patients, respectively; e1a2 is present in 60% to 75% of patients with ph-positive ALL, but only can be detected in 1% CML patients^6^.

So far, only a small number of B-ALL cases with e1a3 BCR-ABL fusion transcript have been reported. The e1a3 BCR-ABL variant is produced by the fusion of BCR exon 1 to ABL exon 3. The presence of this translocation has been associated with a slowly clinical course, low white blood cell count, long-term chronic phase, and good response to treatment in CML^7^,^8^. However, the number of cases is small and the same results were not obtained in ALL. It has been confirmed that the src homology 3 (SH3) domain which was encoded by ABL exon 2 is crucial to the development of BCR-ABL associated leukemia. But the ABL exon 2 region is absent in e1a3 BCR-ABL variant fusion transcript, and only partially encodes the SH3 domains. Therefore, the role of the SH3 domain in ela3 BCR-ABL leukemia is controversial. It can enhance the transformation ability of tyrosine kinase and activate the STAT5 signaling pathway, leading to the occurrence of leukemia. Lacking of SH3 region, the e1a3 transcript patients might appear relatively benign clinical process according to weaken the proliferation and tissue invasion of leukemia cells^9^,^10^. Another view considered the e1a3 and e1a2 transcripts have a similar molecular weight and probably have a similar clinical profile, such as extramedullary infiltration and disease acceleration. overall, its role is still unclear. Miyashita N et al.^11^ reported 17 patients with e1a3 BCR-ABL fusion gene in ALL, and 8 of the 13 available karyotypes showed complex chromosomes which is consistent with the patient results we reported, suggesting that this type may have poorer prognostic outcomes.

Theoretically, all atypical BCR-ABL transcripts should be responsive to TKI-targeted therapy, regardless of BCR sequences fusing to ABL exon 2 or 3, because they all encode the ATP binding pocket (the imatinib binding site) of the ABL kinase. Burmeister et al.10 screened 1214 patients with Ph-positive B-ALL, five of whom were of e1a3 variant. Three of the five patients were treated with TKI and showed treatment reaction, another two patients who did not receive TKI died at 6 and 16 months after diagnosis, respectively. Sun et al.^12^ reported a patient whom simultaneous has different BCR-ABL transcripts named e1a3, e1a4, and e1a5, he achieved short complete remission but relapsed soon after treatment with TKI. This suggested that B-ALL patients with e1a3 transcripts were effective against TKI therapy. This case we reported also showed early response to TKI, but unfortunately it may not overcome the poor prognosis.

According to previous reports, the incidence of e1a3 transcript in BCR-ABL positive ALL patients is 1%-2%. And there has no similar reports in China. Qin et al.^13^ analyzed 4750 CML patients in China and only found 1 patient with e1a3 fusion gene. It may be related to the false negative results which are produced due to the imperfect detection methods. At present, most of the primers contain all the possible breakpoints of BCR gene, while the fracture site of ABL gene is relatively constant in intron 1, so it is easy to ignore in the design of primers. Therefore, it is necessary to design reasonable PCR primers and apply sequencing methods to improve the detection rate of rare BCR-ABL fusion genes. To monitor and quantify these atypical BCR- ABL transcripts, Bhreathnach et al.^14^ suggested using droplet digital PCR (ddPCR) which can divide the sample into multiple independent PCR reactions, improving the effective concentration, and does not need a calibrated standard curve, so as to achieve the absolute quantification of the target molecule.

Mutations in ABL kinase region are an important cause of relapse and treatment failure in Ph-positive ALL, which can inhibit or weaken the binding ability and activity of TKI^15^, and these mutations are prone to be combined with complex karyotypes, suggesting that high heterogeneity and instability of the cytogenetics are more likely to lead to mutations in the ABL kinase region. In Ph-positive ALL, ABL kinase mutations mainly include E255v, T315I, Y253, etc. T315I mutation has the highest frequency among these and is associated with rapid disease progression and worse prognosis^16^,^17^, and it is resistant to TKI drugs such as Imatinib dasatinib, but the third-generation TKI such as ponatinib, olverembatinib [a4] can overcome this mutation^18^. Shin Sy et al.^19^ reported a B-ALL patient with e1a3 variant who received imatinib combined with chemotherapy but failed to achieve complete remission, and subsequently developed the E255v mutation in the ABL kinase region at 12 months and died of multiple organ failure at 16 months. Dong y et al.^20^ reported a Ph-positive ALL patients with a e1a3 BCR-ABL fusion gene and T315I mutation, the disease progressed rapidly, taking only one month from relapse to death. Sheets et al.^21^ reported a patient with e1a3 BCR-ABL fusion transcript in acute myeloid leukemia, he received dasatinib therapy and achieved MRD+ CR after 3 months diagnosis, but relapsed with a T315I mutation and again achieved CR on ponatinib, while subsequently relapsed and died of progressive AML. Our patient had both E255v and T315I mutations which suggested that this rare e1a3 fusion genotype may be more prone to ABL kinase mutation especially T315I, and should first consider the third-generation TKI as a targeted therapy option. Because it may achieve longer complete remission, and reduce the number of relapses, and provide more possibilities for subsequent allogeneic hematopoietic stem cell transplantation. [a5] However, the high cost of the third generation of TKI may limit its application.

## Conclusion

The clinical significance of e1a3 fusion gene remains undermined, we can learn from this rare case that Ph-positive ALL expressing e1a3 BCR-ABL transcript has a poorer prognosis and is more likely to have mutations in the ABL kinase region such as T315I, and third-generation TKI and allo-HSCT or other new treatments should be considered as the initial treatment. Reporting this case is useful to identification the characteristics of e1a3 BCR-ABL ALL and may provide optimal treatment strategies for patients with this rare genotype. In the future, with further research of this subtype of disease, I believe that more new drugs or treatments can be discovered to achieve a better prognosis for this group of patients.

## Data Availability

The data presented in this case report are available on request from the corresponding author.
